# Topics of the Month

**Published:** 1896-06

**Authors:** 


### TOPICS OF THE MONTH.

A Retrospect.—Fifty-six women have graduated from the Medical Department of the University of Buffalo in the last twenty years. Dr. Mary B. Moody was the first to receive her diploma, graduating in the Centennial year. Until 1890 but two women had been honor students, though others had ranked high. Since that time thirteen women, out of a total of thirty-six graduating, have taken honors; twice a woman has led her class.

The full list of women graduates, as compiled from the Alumni catalogue, issued by the University, is as follows:

*Kathryn M. Bailey, Buffalo, '89; Gertrude E. Beebe, '91; Ida C. Bender, 671 Ellicott street, Buffalo, '90; Alice McL. Ross Bennett, 191 Delaware avenue, Buffalo, '90; Clara E. Bowen, 158 Benzinger street, Buffalo, '92; Marie L. Benoit, Montreal, '96; Ava M. Carroll, '88; Evangeline Carroll, 285 Ashland avenue, Buffalo, '93; Jane Wall Carroll, 285 Ashland avenue, Buffalo, '91; Martha F. Caul, 936 West avenue, Buffalo, '91; N. Victoria Chappell, 326 West Ferry street, Buffalo, '92; Isabel A. Church, B. S., 210 W. 133d street, New York, '93; Salina P. Colgrove, Ph. G., Salamanca, N. Y., '88; Amanda M. Congdon, Cuba, N. Y., '92; *Annie B. Culver, Des Moines, Ia., '84; Mary I. Denton, 228 Potomac avenue, Buffalo, '91; Mary E. Dickinson, Dansville, N. Y., '90; Louise Downer, Warsaw Sanitarium, Warsaw, N. Y., '86; Ella May Doyle, East Concord, N. Y., '93; Amelia Dresser, 92 Chester street, Buffalo, '93; Alice B. Foster (Bryn Mawr), 57 Vernon street, Wakefield, Mass., '91; Jane North Frear, 28 Orton Place, Buffalo, '94; Maud J. Frye, 224 Allen street, Buffalo, '92; Anna Wadsworth Hatch, Sank Cen., Minn., '89; Jeannette Potter Himmelsbach, 137 West Tupper street, '90; Mary M. Huntley, 369 Hudson street, Buffalo, '96; Elizabeth Johnson, 68 West 38th street, New York, '87; Sophia B. Jones, Six Lakes, Mich., '83; Rachel J. Kemball, 228 West Tupper street, Buffalo, '84; Regina F. Keyes, 298 Bouck avenue, Buffalo, '96; Elizabeth M. King, Akeley Inst., Grand Haven, Mich., '93; Ada C. Latham, 174 Dodge street, Buffalo, '92; Cora Billings Lattin, 212 Humboldt Parkway, Buffalo, '94; Emma C. LeFevre, 558 East Church street, Elmira, N. Y., '92; Elizabeth Fear Leffingwell, Summit, N. J., '88; Emma L. McCray, Lovell's Station, Pa., '91; Jennie L. Messerschmidt, Bath, N. Y., '93; Mary Blair-Moody, Fair Haven Heights, New Haven, Conn., '76; Helen Kennedy Morehouse, 183 Jewett avenue, Buffalo, '85; Nellie Edmunds Murray, 153 Clinton street, Tonawanda, N. Y., '92; Sarah H. Perry, 103 South Fitzhugh street, Rochester, N. Y., '82; Lillian Craig Randall, 502 Elmwood avenue, Buffalo, '91; Mary E. Runner-Sanford, Buffalo, '81; Sarah E. Simonet, Croghan, N. Y., '85; Mary Jane Slaight, 33 Chestnut street,

* Deceased.

Rochester, N. Y., '80; Ellen Roberts Spragge, 426 Connecticut street, Buffalo, '88; Elizabeth M. Squier, Albion, N. Y., '93; Isabella H. Stanley, 415 Central avenue, Dunkirk, N. Y., '83; Anna M. Stuart, Elmira, N. Y., '95; Clara B. Talbot Weidman, Rockport, Me., '90; Marian A. Townley, 14 Aurora street, Ithaca, N. Y., '89; Amelia Earle Trant, 1268 Main street, Buffalo, '94; Bina Potter-Van Denbergh, Dansville, N. Y., '83; Stella Cox Venable, Geneseo, N. Y., '88; *Frances Weidman Wynds, Brooklyn, '91; Mary Berkes Wetmore, 30 Woodlawn avenue, Buffalo, '80.

---

THE Legislature of 1895-6 has numerous sins of omission and commission to be laid at its door. Of these, the one that has created the most unfavorable comment from members of the medical profession in all quarters of the state is a bill passed late in the session and referring to the management of the state hospitals for the care of the insane. In its original form it was a bill to provide for a codification of the laws relating to lunacy, but an amendment which was quietly added makes it a most dangerous measure, giving to the Governor the power to turn out of office every trustee or manager of every state hospital in New York, excepting those of the Middletown (Homeopathic) Hospital! As there has been no charge of incompetency or of any abuses preferred, there can be but one inference drawn from such an unprecedented proceeding—the places are needed as political prizes! It was hoped that Governor Morton would refuse to sign this astonishing, infamous bill, but—*Que voulez-vous ?*

---

UNDER the title, "An act to regulate the employment of women and children in mercantile establishments, and to provide that the same shall be enforced," the last Legislature passed and the Governor approved a bill of interest not only to the general public but to the medical profession also. The most noteworthy features of this act are the regulation of the number of hours a week that women and children can lawfully be employed, the sanitary arrangements of the places in which they do their work, particularly with reference to light, ventilation, the location of water-closets, and the like, all of which must be approved by the local health authorities, and the regulation of the length of the luncheon hour. But the one provision of peculiar interest is found in Section 2, and refers to a system of registration of a certain class of

* Deceased.

employees, the most thorough ever attempted. We quote from this section: "No child under 14 years of age shall be employed in any mercantile establishment of this state. It shall be the duty of every person employing children *to keep a register*, in which shall be recorded the *name*, *birthplace*, *age* and *place* of residence of every person employed by him under the age of 16 years." The bill also makes it obligatory for each child under 16 who is employed to file with his or her employer a certificate obtained from the local Board of Health, stating the date and place of birth, the *color of eyes* and *hair*, the *weight* and *height*, together with any distinguishing facial marks of the individual named in the certificate, and also setting forth the fact that the health commissioner, or any person designated by him, considers such child physically able to perform the work intended. This law is to take effect September 1, 1896. It is made incumbent upon local health boards to see that the law is faithfully observed, and penalties, consisting of fines or imprisonment, or both, are fixed for omissions or violations of the law. It is obvious that if the foregoing law is to be enforced in Buffalo in a conscientious manner, the work of the health department will be greatly increased and additional health inspectors must be provided. The work to be done under this law is of a kind that women physicians are peculiarly well fitted to do satisfactorily. We hope that our excellent commissioner will not overlook this fact when he sets his department at work in obedience to the law.

---

In the report of 1895 of the New Hospital for Women, in London, is incorporated the following personal: "It may interest our supporters to learn that Miss Ellaby, M. D., ophthalmic surgeon to the hospital, was requested to go to India last October to operate on the Maharanee of Jamnagar for cataract. The patient was 70 years of age and for five years had perception of light only. Both eyes were operated upon with a month's interval between the two operations; the recovery in both cases was without complication of any kind, and the sight of both eyes was completely restored."

---

The students of the London School of Medicine for Women, which numbers 150, receive in the wards and out-patient department of the Royal Free Hospital their clinical instruction. The Royal Free was opened in 1828, and for the past eighteen years has afforded women medical students advantages without which they would not be eligible to go up for examination by the various examining boards. As to their success in examinations, Mr. Thies, the secretary of the Royal Free, said: "Seeing that in one year nine of them went in for the London M. B. and all passed, I think I may say they were successful." On July 22, 1895, the formal opening occurred by the Prince and Princess of Wales of the new front building of the Royal Free, which completed the reconstruction commenced forty-three years since. Kings George IV. and William IV., the Duchess of Kent and the Queen—soon after her ascension to the throne—were patrons, and her Majesty has ever since taken a lively interest in the prosperity of the institution.

---

THE following comment, signed "Editor," is found appended to a correspondent's letter in the *London Lancet* of November 9, 1895. We call the attention of dress reformers to it: "We have at different times spoken our minds concerning tight lacing and the misshapen corset. But the well-fitting corset is a valuable article of attire. With different modifications it becomes, as all our profession know, a useful surgical instrument; and the ordinary corset provides a grateful support in many conditions."

---

ACCORDING to the last report of the Commissioner of Education there were, during the year 1892-3, 1,302 women enrolled as students in the various medical schools of the United States. Of these 827 were in the regular and 330 in the homeopathic schools. During the same year there were 98 women studying pharmacy and 63 studying dentistry. The report states that there are in this country seven schools for the medical education of women exclusively. Of the 104,803 physicians and surgeons in the United States, 4,555, according to the census of 1890, were women.

---

A BILL has passed the Legislature of Virginia authorising the appointment of female physicians for the female wards of the State insane asylums.

---

NOTWITHSTANDING the protest of numerous medical and scientific societies, the Committee on the District of Columbia has reported a bill to restrict vivisection in the district. The American Medical Association in session at Atlanta, May 5–8, adopted resolutions earnestly protesting against the passage of the bill, truthfully observing that far more unnecessary pain is constantly being inflicted upon the lower animals for sport and for game than in biologic and pathologic laboratories.

